# Pharmacologic IRE1α kinase inhibition alleviates aortic dissection by decreasing vascular smooth muscle cells apoptosis

**DOI:** 10.7150/ijbs.63593

**Published:** 2022-01-01

**Authors:** Wenjun Zhang, Mengwen Wang, Kun Gao, Xiaodan Zhong, Yang Xie, Lei Dai, Wanjun Liu, Yujian Liu, Xingwei He, Shiliang Li, Thati Madhusudhan, Hongjie Wang, Hesong Zeng

**Affiliations:** 1 Division of Cardiology, Department of Internal Medicine, Tongji Hospital, Tongji Medical College, Huazhong University of Science and Technology, Wuhan, 430030, PR China.; 2 Hubei Key Laboratory of Genetics and Molecular Mechanisms of Cardiological Disorders, Wuhan, 430030, PR China.; 3 Division of Cardiothoracic and Vascular Surgery, Tongji Hospital, Tongji Medical College, Huazhong University of Science and Technology, Wuhan, Hubei, China.; 4 Center for Thrombosis and Hemostasis, University Medical Center Mainz, Langenbeckstr. 1, 55131 Mainz, Germany.

**Keywords:** Aortic dissection, Endoplasmic Reticulum Stress, X-Box binding Protein 1, IRE1α, Reactive Oxygen Species, Apoptosis

## Abstract

Aortic dissection (AD) is a rare but catastrophic disorder, and associated with significant morbidity among survivors. This study aimed to target IRE1α-XBP1s pathway pharmacologically, and evaluate its therapeutic potential in the occurrence and progression of AD. Western Blot and immunohistochemistry results showed that expression of XBP1s was significantly increased in the human aorta samples of AD group in compared with the control group, and exclusively in aortic vascular smooth muscle cells (VSMCs). Further *in vitro* study revealed that Angiotensin II (Ang II) could increase the expression of XBP1s and promote its nuclear translocation in cultured VSMCs, which leads to numerous gene transcription, including *gp91phox, Chop, Cleaved-caspase 3, Bax, and Bcl-2*. These genes contribute to the production of reactive oxygen species (ROS), VMSCs phenotypic switch and apoptosis. Whereas an IRE1α endoribonuclease domain inhibitor MKC-3946 could reverse it. Finally, the efficacy of MKC-3946 was tested in a mouse AD model. As shown *in vitro*, MKC-3946 could reduce the expression of XBP1s and protect against AD by suppressing XBP1s associated ROS production and apoptosis in VSMCs *in vivo*. The current study revealed the relevant role of IRE1α-XBP1s signaling pathway in AD occurrence and progression. MKC-3946 could be of great potential in clinical application.

## Introduction

Aortic dissection (AD) is a rare but catastrophic disorder and it is associated with significant morbidity among survivors. Open surgery remains the standard for treatment, while endovascular aortic repair (EVAR) has been shown to be feasible and encouraging in selected patients 1, 2. However, both treatments require sophisticated facilities and skilled specialists, which hampers their wide application, especially in underdeveloped regions. Thus, it is urgent to develop an alternative and supplementary medication that could be an effective therapy or a pre-operation bridging approach. Since the detailed molecular mechanisms involved in AD formation and progression are poorly understood, there is so far no reliable pharmacological prophylactic measures.

The key histopathologic feature of AD is medial degeneration, which is characterized by the vascular smooth muscle cell (VSMC) contractile phenotype loss and depletion, and extracellular matrix degradation 3. Despite extensive investigation, the underlying molecular mechanism(s) contributing to VSMC dysfunction, and AD formation and progression remains largely elusive 4. Endoplasmic reticulum (ER) stress has been involved in the pathogenesis of aortic aneurysm 5, 6. There are 3 pathways in the ER-stress response (unfolded protein response, UPR): the PERK (protein kinase R-like ER kinase), ATF-6 (activating transcription factor 6), and IRE1α (inositol-requiring enzyme 1α) pathways. Higher gene and protein expression of ATF6, IRE1α, XBP-1, C/EBP-homologous protein (CHOP) were detected in human aneurysmatic samples 6. Further study showed that significant VSMC loss, excessive apoptosis and ER stress, along with inflammation, were present in AD aorta samples from both mouse and human, which demonstrated that ER stress promotes VSMCs inflammation, degeneration and apoptosis 5. Under ER stress condition, IRE1α is activated by oligomerization and autophosphorylation, resulting in activation of its endoribonuclease to cleave and initiate splicing of the *XBP1*mRNA. A 26-nucleotide intron from *XBP1* mRNA is removed by activated IRE1α endoribonuclease, resulting in a translational frame-shift to modify unspliced XBP1 (XBP1u: inactive) into spliced XBP1 (XBP1s: active) 7. XBP1s then translocates to the nucleus and transcriptionally regulates genes that are involved in protein folding, ER-associated degradation (ERAD), glycosylation, autophagy, lipid biogenesis, *etc.* 4, 8 Accumulating data has suggested an essential role of XBP1 splicing in angiogenesis, endothelial proliferation, and apoptosis and in VSMCs migration during atherosclerosis and post injury neointima formation 9-11. Especially, constitutive knockout of XBP1 results in embryonic lethality at embryonic day (E) 13.5 and leads to vessel malformation, which suggests a vital role for XBP1 in vascular homeostasis 9.

In this study, using a small-molecule inhibitor, we targeted IRE1α-XBP1s pathway in VSMC cell line *in vitro* and in an angiotensin II induced AD mouse model *in vivo*. We hypothesize that inhibition of XBP1 splicing will protect against AD formation and progression by preserving VSMC homeostasis. These studies provide the preclinical basis for developing effective preventative and pharmacological treatment for AD.

## Materials and Methods

### Animal experiment

The animal procedures conform to the guidelines from Directive 2010/63/EU of the European Parliament on the protection of animal used for scientific purpose and approved by the Institutional Animal Research Committee of Tongji Medical College. ApoE^-/-^ mice were purchased from Beijing Vital Laboratory Animal Technology Co., Ltd. ApoE^-/-^ mice (C56BL/6J background) were housed at the animal care facility of Tongji Medical College under specific pathogen-free conditions and fed a normal diet. The mouse AD model was established according to our previous published protocol 12. In short, 8-week-old male ApoE^-/-^mice were given β-aminopropionitrile (BAPN) at a concentration of 0.1% for 3 weeks and infused *via* osmotic mini pumps (Alzet, Cupertino, CA) with either saline or 2,500 ng/kg/min angiotensin II (Ang II) (Sigma-Aldrich, St.Louis, Mo) for 2 weeks. To evaluate the effect of IRE1α inhibitor MKC-3946 treatment on AD initiation and progression, treatment was initiated 2 weeks during Ang II infusion. MKC-3946 was freshly prepared in PBS and administered to mice at a dose of 100 mg/kg every day through intraperitoneal injection as described elsewhere 13.

### Cell culture

Vascular smooth muscle cells (VSMCs) (ATCC, Manassas, VA) were cultured in DMEM containing 10% bovine serum (Gibco, Grand Island, NY) under 37℃ and 5% CO2 condition. In the Ang II group, cells were incubated with 1.0 μM Ang II (Sigma-Aldrich, St. Louis, MO) for 24 hours. For intervention study, 40 μM MKC-3946 (Selleck, CA) were added before Ang II treatment.

### Western blot

Total proteins were extracted using RIPA. Protein concentration was determined by the BCA Assay Kit (Boster, Wuhan). After boiling for 5 min, equal amounts of protein (20 μg) were added in 10% SDS-PAGE gels for electrophoresis at 70V for 2.5 hours, followed by transfer to PDVF membranes (Milipore, Billerica, MA) at 200 mA for 2 hours. The membranes were then blocked by 5% Bovine Serum Albumin in TBST (1×, pH=7.4) and incubated at 4℃ overnight with one of following primary goat anti rabbit antibodies: XBP1s, ATF4, ATF6, CHOP, GRP78, IRE1α, p-IRE1α, Caspase3, Bax, MMP2, MMP9, SM22α, α-SMA, Calponin and Bcl-2. Membranes were incubated with peroxidase-conjugate secondary antibody IgG (1:5000). Protein bands were detected by electrochemiluminescence system and analyzed by Image J software. Samples were normalized by corresponding measures of GAPDH derived from the same samples in each blot. All experiments were performed at least three times.

### Histology, immunohistochemistry and immunofluorescence

All mouse and human aortae were fixed in 4% paraformaldehyde for 48 hours, embedded in paraffin, and sliced into 4 μm sections. Histomorphology was evaluated by hematoxylin and eosin (H&E) staining. Aortic wall elastin integrity was assessed by EVG staining. Images were captured using an OLYMPUS BX53 Microscope (OLYMPUS, Beijing, China). Immunohistochemistry was observed using an OLYMPUS BX53 Microscope (OLYMPUS, Beijing, China) after XBP1s (1:100), ATF6(1:100), CHOP (1:100), Cleaved-caspase3(1:100), MMP2 (1:100), MMP9 (1:100) and gp91(1:100) staining and was analyzed using Image J software. Immunofluorescence was observed after XBP1s (1:100)/α-SMA (1:500) staining and then 4',6-diamidinao-2-phenylindole (DAPI) was used to counterstain the nucleus.

### DHE staining assay

DHE assay kit (Beyotime, China) was used to detect the intracellular ROS levels. Briefly, cells were pre‐treated with MKC-3946 (40 μM) for 30 min and then treated with Ang II (1.0 μM) for 24 hours. Then, they were tested by a fluorescent probe (1:1000) and incubated at 37℃ for 30 min after washed and centrifugation carefully. The fluorescence of DCF was measured using a Fluorescence microscope (OLYMPUS, Beijing, China). And the mean fluorescence intensity was quantitative analyzed by Image J software.

### TUNEL staining

TUNEL kit (Beyotime, China) was used to detect tissue apoptosis. Proteinase K working solution was diluted with PBS at a final concentration of 20 µg/ml. Each section was treated with 20 µl of proteinase K working solution at room temperature (RT) for 10 min. After rinsing with PBS for 2-3 times, each section was incubated with 100 µl of 1× equilibration buffer for 10-30 min at RT, followed by addition of a solution containing 50 µl of TdT enzyme. Then, the sections were incubated at 37 °C for 60 min. The nuclei were counter-stained with 0.1% DAPI solution.

### RT-PCR

To examine the downstream UPR genes in VSMCs, we performed UPR pathway-specific gene expression analyses. Quantitative RT-PCR were conducted essentially as previously described 14. PBS washed VSMCs were placed on ice and dissolved in TRIZOL (Life Technologies, Shanghai, China) for isolation of total RNA following the manufacturer's protocol. Quality of total RNA was ensured on an agarose gel and by analyses of the A260/280 ratio. The reverse transcription reaction was conducted with 1 μg of total RNA using the Super Script reagents and oligo (dT) primers (Sangon Biotech, Shanghai, China). cDNA was amplified using the primers listed in the **Table 1**.

### Statistical analysis

All results were obtained and confirmed in at least three independent experiments. Data were calculated and presented as means ± SD. One-way analysis of variance and the Student *t* test were used for calculating statistical significance by Graphpad Prism 7.0 (GraphPad Software, San Diego, CA). *P*<0.05 were considered significant.

## Result

### XBP1s was significantly increased in aortic vascular smooth muscle cells of acute aortic dissection patient

**Figure 1:**
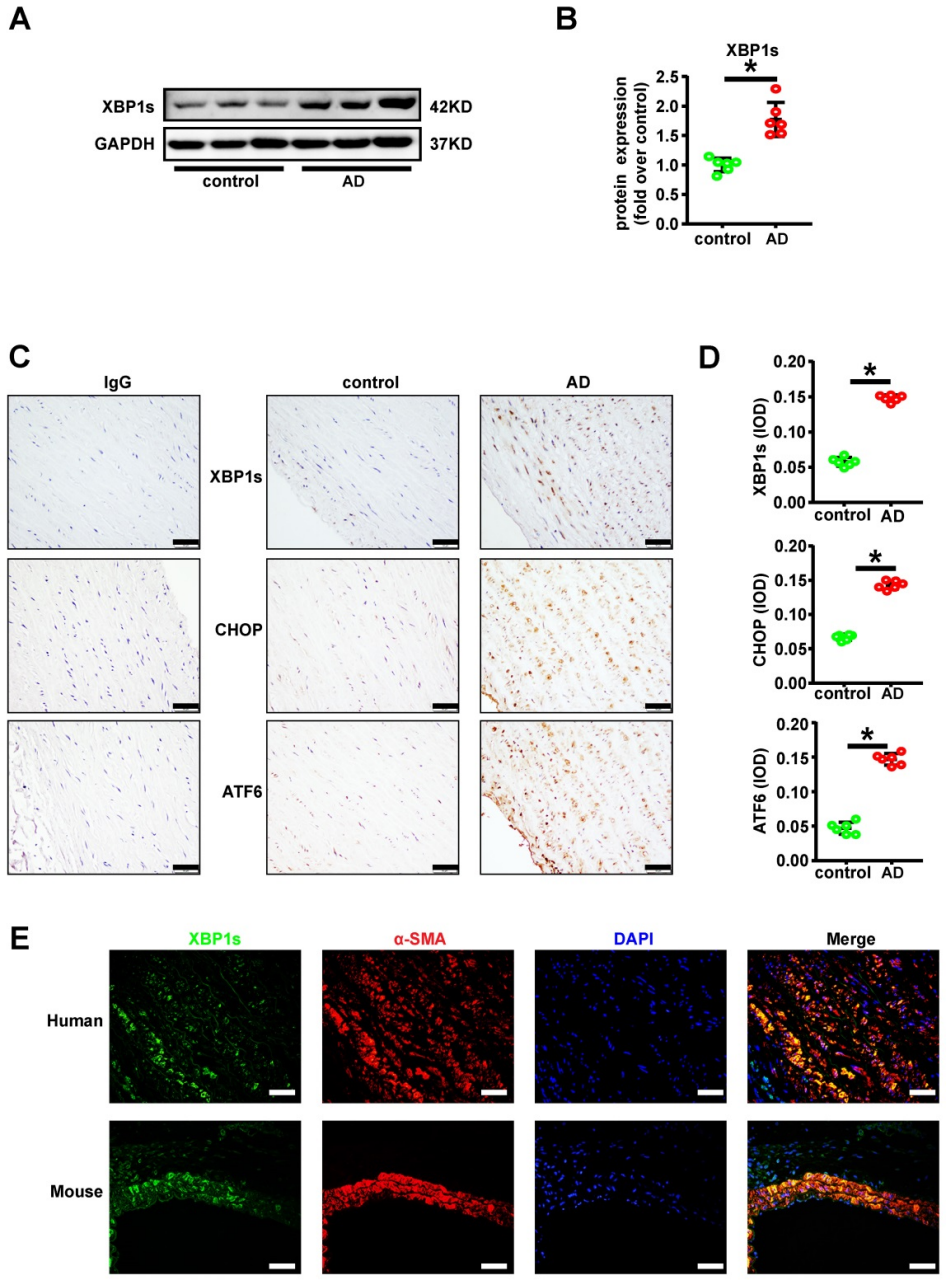
XBP1s was significantly increased in vascular smooth cells of human aorta with acute aortic dissection (AD). **(A-B)** Expression of XBP1s in aortae of normal and AD patient is evaluated by western blot and summarized by scatter plot. **(C-D)** Immunohistochemistry staining shows the expression of XBP1s, CHOP and ATF6 in human AD aorta tissues compared with respective control aortae. **(E)** Double-labeled immunofluorescence assay was used to detect expression of XBP1s in human and mouse aortae. All data represent the means ± SEM; * *p* < 0.05 *vs.* control; Scale bar=50 μm; n=6/group.

Convincing studies have implied an essential role of XBP1 splicing during atherosclerosis and post injury neointima formation 9-11. To explore the role of XBP1s in acute aortic dissection (AD), we determined the relative XBP1s protein expression with Western Blot in the human aorta samples. We found that the XBP1s' expression level was significantly higher in aortic samples of AD patient in compared with the healthy controls (**Figures 1A-B**). At the same time, immunohistochemistry staining of XBP1s on the paraffin embedded aorta sections further suggested the higher expression pattern and intriguingly revealed vascular smooth muscle prone staining of XBP1s, as well as other ER stress key effectors such as CHOP and ATF6 (**Figures 1C-D**). In order to determine the cell type specific expression of XBP1s, double-labeled immunofluorescence assay was performed. As we assumed, the expression of XBP1s was exclusively colocalized with vascular smooth muscle marker α-SMA in human and mice with AD (**Figure 1E**). These data indicate that within aorta of AD, the expression of XBP1s is predominantly induced in the vascular smooth muscle cell.

### IRE1α endoribonuclease domain inhibitor MKC-3946 decreased XBP1s, CHOP, ATF6 and ATF4 expression *in vitro*

**Figure 2:**
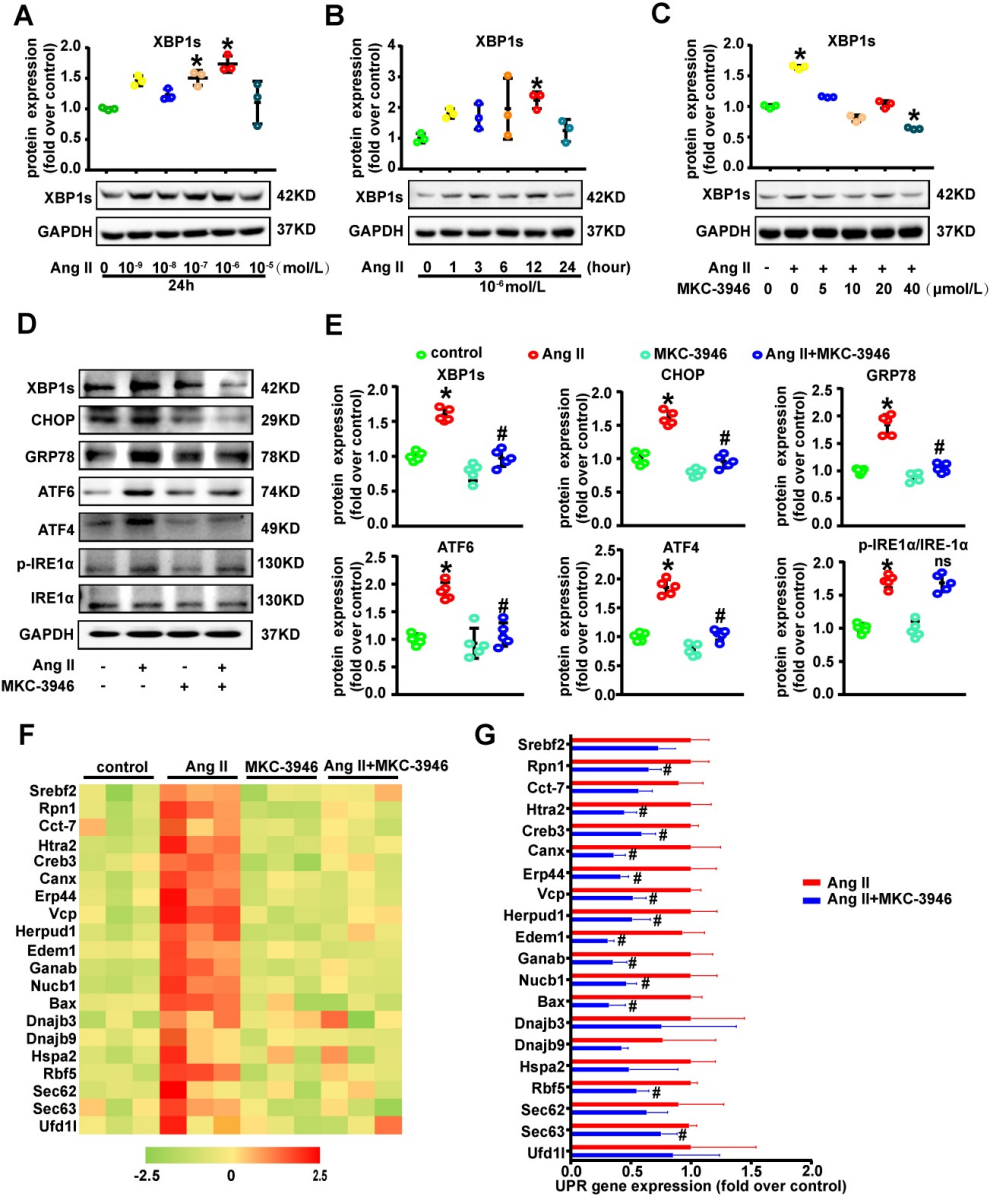
IRE1α endoribonuclease domain inhibitor MKC-3946 decreased XBP1s, CHOP, ATF6 and ATF4 expression* in vitro*. **(A-B)** Western blotting shows the expression of XBP1s was induced by Ang II in a concentration and time dependent manner.** (C)** Western blotting shows IRE1α endoribonuclease domain inhibitor MKC-3946 can suppress the expression of XBP1s induced by Ang II in a concentration dependent manner. VSMCs were preincubated with MKC-3946 in various concentration for 30 min and then treated with Ang II (1.0 μM) for 12 hours. **(D-E)** Western blotting analysis of ER stress related protein in VSMCs. VSMCs were preincubated with MKC-3946 (40 μM) for 30 min and then treated with Ang II (1.0 μM) for 12 hours. MKC-3946 treatment can decrease XBP1s, CHOP, ATF6 and ATF4 expression. **(F)** Heat map summarizes enrichment of condition-specific concordant UPR genes.** (G)** And their impact on regulation of UPR-gene expression. Bar graph summarized the results. At least three independent experiments were performed. All data represent the means ± SEM; * *p* < 0.05 vs. control group; # *p* < 0.05 vs. Ang II group; One-way ANOVA.

Given the observation that XBP1s expression is induced in AD, we hypothesized that XBP1s participates in the formation and progression of AD. To further study the role of XBP1s in AD *in vitro*, we firstly did the dose and time response experiments for XBP1s expression induced by Ang II in cultured VSMCs and found that Ang II promotes the expression of XBP1s in cultured VSMCs (**Figures 2A-B**). We know that there are 3 pathways in the ER stress response: the PERK (protein kinase R-like ER kinase), ATF-6 (activating transcription factor 6), and IRE1α (inositol-requiring enzyme 1α) pathways 6. On sensing the accumulation of unfolded proteins, IRE1α cleaves unspliced *XBP1* (*XBP1u*) mRNA and removes a 26-nucleotide-long intron, resulting in the production of a spliced *XBP1* (*XBP1s*) 7. When we used IRE1α endoribonuclease domain inhibitor MKC-3946 on cultured VSMCs, the expression of XBP1s induced by Ang II treatment was substantially suppressed in a concentration dependent manner (**Figure 2C**). Simultaneously, we found that MKC-3946 markedly decreased GRP78, CHOP, ATF6 and ATF4 expression *in vitro* (**Figures 2D-E**). However, MKC-3946 treatment did not affect the IRE1α and p-IRE1α expression level in cultured VSMCs (**Figures 2D-E**). These data show that MKC-3946 could effectively suppress the expression of XBP1s and other ER stress pathway effectors provoked by Ang II in cultured VSMCs *in vitro*. To further prove its functional relevance in ER-stress, we examined the downstream XBP1s associated UPR genes in VSMCs that were incubated with Ang II and/or MKC-3946. As demonstrated in **Figures 2F-G**, Ang II treatment promoted XBP1s-dependent UPR gene expression including components of ERAD (*Htra2, Herpud1, Vcp*), protein binding and folding (*Rpn1, Canx, Cct7, Hspa2, Dnajb9, Erp44*), protein ER membrane translocation (*Sec62, Sec63*), cholesterol biosynthesis and lipid homeostasis (*Srebf2*), while MKC-3946 treatment reversed these changes. Besides, there are several genes (*Bax, Creb3, Ganab, Nucb1*) contributing directly to the apoptotic pathway, indicating an important role of VSMC apoptosis in AD.

### MKC-3946 treatment can decrease oxidative stress, regulate phenotypic switch and inhibit apoptosis in VSMC by suppressing nuclear translocation of XBP1s *in vitro*

**Figure 3:**
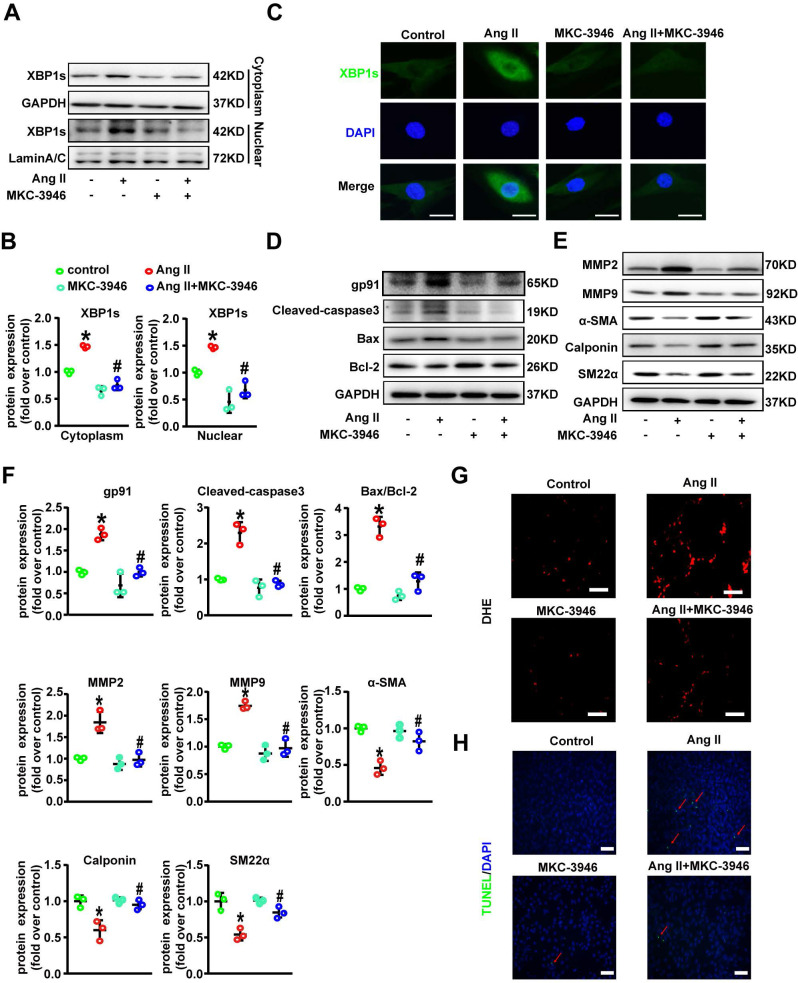
MKC-3946 treatment can improve VSMC contractile phenotype and decrease oxidative stress and apoptosis by suppressing nuclear translocation of XBP1s *in vitro*. **(A-B)** Western blotting shows the expression of XBP1s in cytoplasm and nucleus. MKC-3946 treatment can reduce the expression of XBP1s in cytoplasm and nucleus. **(C)** Cellular immunofluorescence staining shows the distribution of XBP1s in VSMCs. Green represent XBP1s and blue represent nuclear. Scale bar = 10µm. **(D)** Western blotting shows the expression of gp91, Cleaved-caspase3, Bax and Bcl-2 in VSMCs. MKC-3946 can decrease the expression of gp91, Cleaved-caspase3, Bax and increase the expression of Bcl-2. **(E)** Western blotting shows the expression of α-SMA, Calponin and SM22α in VSMCs. MKC-3946 can enhance the expression of α-SMA, Calponin and SM22α. **(F)** Scatter blots summarize results in (E) and (F). **(G)** Representative images of VSMCs showing DHE fluorescence in different groups. Scale bar=50 μm. DHE, dihydroethidium. **(H)** Representative images of VSMCs showing TUNEL fluorescence in different groups. Green represent TUNEL and blue represent nucleus. Scale bar=100 μm. At least three independent experiments were performed. All data represent the means ± SEM; * *p* < 0.05 *vs.* control group; #* p* < 0.05 *vs.* Ang II group; One-way ANOVA.

We next tried to explore the mechanistic link between elevated XBP1s level in VSMCs and AD. Interestingly, western blot results showed that Ang II treatment induced both cytoplasmic and more prominently nuclear levels of XBP1s in VSMCs, which was suppressed by MKC-3946 treatment (**Figures 3A-B**). Moreover, we also tested the distribution of XBP1s in VSMCs by cellular immunofluorescence staining, and the results were consistent with our western blot findings (**Figure 3C**). Ang II has been shown to activate vascular NAD(P)H oxidase and stimulate ROS production in cultured VSMC 14, ROS and ROS induced ER stress can trigger the apoptosis pathway 15. Previous studies have shown that the switch from a contractile phenotype to a synthetic one in VSMC plays an important role in AD formation and progression 16-18. A synthetic VSMC phenotype represents a dedifferentiated form characterized by increased proliferation, migration and extracellular matrix synthesis in concert with decreased expression of contractile markers, which results in maladaptive phenotype alterations that ultimately lead to a proinflammatory state and VSMC apoptosis 19, 20. Interestingly, oxidative stress is proved to be a major contributor to this phenotypic switch 21, 22. Furthermore, Ang II elicits many of its (patho)physiological actions by stimulating ROS generation, and it has been reported to induce the synthetic phenotypic switching of VSMC and promote AD formation and progression 14, 23. Thus, we hypothesize that Ang II can contribute to AD formation and progression *via* ROS triggered VSMC synthetic phenotypic switch and aortic wall homeostasis impairment. Therefore, we next examined the activation status of ROS-associated NAD (P) H subunits gp91 and Cleaved-caspase3, Bax and Bcl-2 in VSMCs. Additionally, we also used dihydroethidium staining to detect the location of superoxide production in VSMCs. We found that MKC-3946 treatment can markedly decrease the expression of gp91, Cleaved-caspase3, Bax and increase the expression of Bcl-2 induced by Ang II in cultured VSMCs (**Figures 3D, F**). Besides, MKC-3946 treatment could significantly reduce the ROS production in cultured VSMCs *in vitro*, as illustrated by the dihydroethidium staining (**Figure 3G**). Subsequently, MKC-3946 treatment could partially reverse the synthetic phenotypic switch of VSMC induced by Ang II, as reflected by the upregulation of specific molecular markers, including SM22α, α-SMA and Calponin. Simultaneously, the Ang II provoked expression of MMP2 and MMP9 by VSMC was significantly suppressed by MKC-3946 treatment (**Figures 3E-F**). Besides, we conducted the TUNEL fluorescent staining experiment and found that MKC-3946 treatment could significantly alleviate the apoptosis of cultured VSMCs induced by Ang II (**Figure 3H**). Taken together, these results suggest that Ang II induced oxidative stress, phenotypic switch and apoptosis in VSMCs is highly dependent on nuclear translation of XBP1s and could be alleviated by MKC-3946 treatment* in vitro*.

### MKC-3946 treatment alleviated Ang II-induced AD formation and the destruction of the middle layer of aortae in ApoE^-/-^ mice *in vivo*

**Figure 4:**
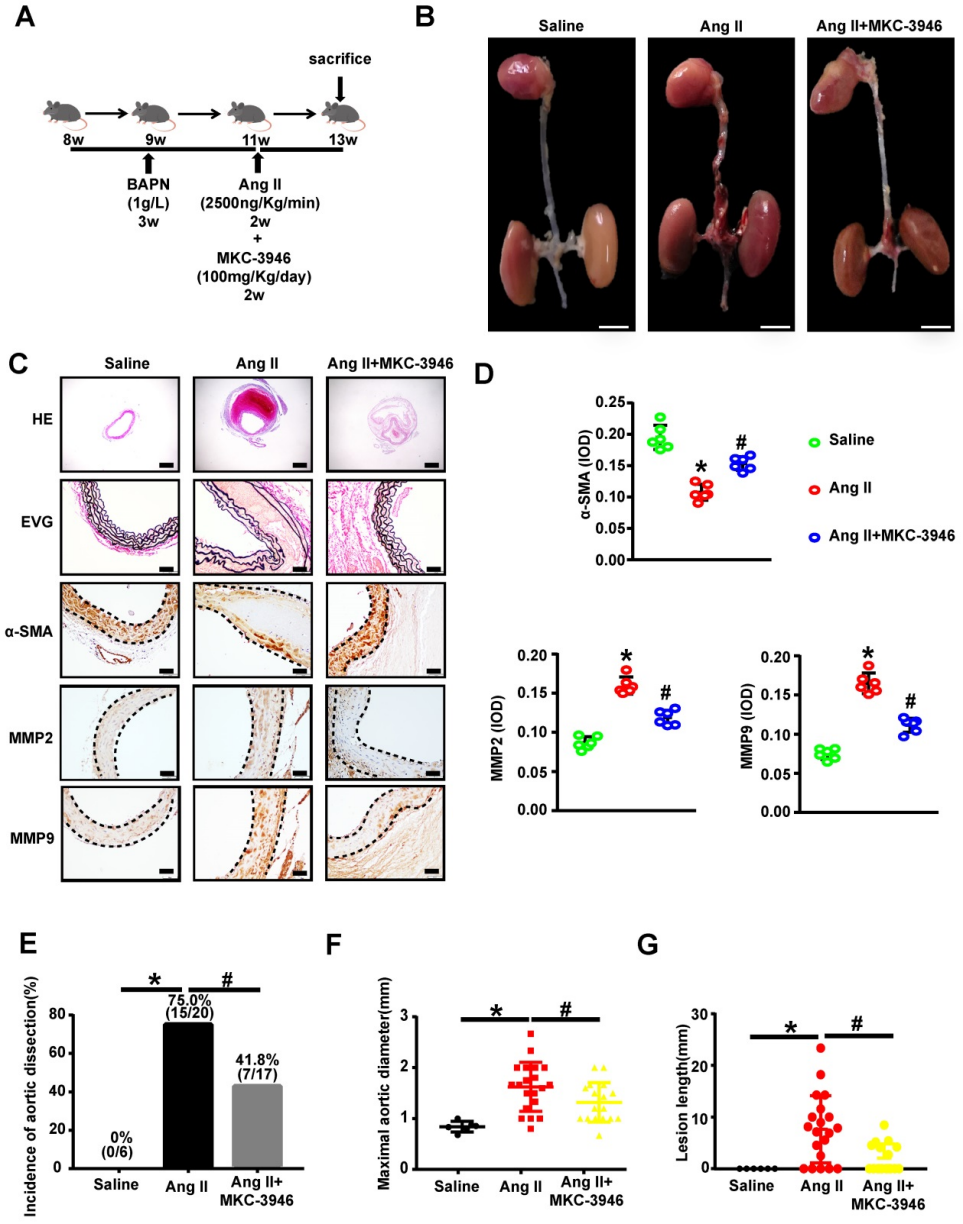
MKC-3946 treatment alleviated Ang II-induced AD formation and the destruction of the middle layer of aortae in ApoE^-/-^ mice. **(A)** Schematic illustration of the AD experimental mouse model. 8- week-old ApoE^-/-^ male mice were given β-aminopropionitrile (BAPN) at a concentration of 0.1% for 3 weeks at first and infused with saline or Ang II (2500 ng/kg/min) *via* subcutaneous osmotic minipumps for 2 weeks. One group received MKC-3946 100 mg/kg/day intraperitoneally at the same as the Ang II osmotic minipump was implanted. **(B)** Representative images of aortae isolated from ApoE^-/-^ mice in different groups.** (C)** H&E, α-SMA, MMP2, MMP9 immunohistochemical and elastin van Gieson staining of aortae with different interventions are shown. MKC-3946 treatment significantly alleviated Ang II induced aortic elastin degradation in the media and stabilized the aortic wall. **(D)** Quantitative analysis of α-SMA, MMP2 and MMP9. **(E)** MKC-3946 treatment significantly reduced the incidence of AD. **(F-G)** MKC-3946 treatment significantly decreased the AD lesion lengths and maximal abdominal aortic diameter induced by Ang II infusion. All data represent the means ± SEM; n=6 mice in saline group, n=20 mice in Ang II group, n=17 in Ang II+MKC-3946 group; * *p* < 0.05 *vs.* saline group; #* p* < 0.05 *vs.* Ang II group; One-way ANOVA.

To explore the potential therapeutic role of MKC-3946 *in vivo*, we applied an AD mouse model. In this model 8-week-old ApoE^-/-^ mice were given BAPN (1.0 g/L) into drinking water for 3 weeks and then infused of Ang II *via* osmotic minipumps for 14 days, which has been repeatedly demonstrated by us and other groups to cause aortic dissection pathology 12 (**Figure 4A**). To assessed the histological features of the AD, we performed H&E, α-SMA, MMP2, and MMP9 immunohistochemical and elastin staining. Histologic examination revealed marked enlargement of the luminal area, upregulation of MMP2/9 expression, destruction of the media and marked thinning of the adventitia in the Ang II group. Surprisingly, these changes were significantly alleviated by MKC-3946 treatment (**Figures 4B-D**). Meanwhile, we also found that Ang II-infusion resulted in an incidence of AD (75.0%, 15/20) in ApoE^-/-^mice and resulted in a higher maximal aortic diameter (1.624±0.107 mm) in ApoE^-/-^ mice than in saline group (0.8405±0.04282 mm). MKC-3946 treatment markedly decreased the incidence of AD (41.8%, 7/17) and the maximal aortic diameter (1.308±0.08902 mm) induced by Ang II (**Figures E-F**). Similarly, the length of aortic wall destruction due to AD was significantly greater in the aortic arch, descending thoracic aorta, and total aorta in the Ang II group compared with the saline group, while MKC-3946 treatment can significantly alleviate it (**Figure 4G**). These dates indicated that MKC-3946 treatment could protect against Ang II-induced AD formation and the destruction of the middle layer of aortae *in vivo* and further showed that XBP1s plays a vital role in the formation and progression of AD.

### MKC-3946's protective effect on AD depend on XBP1s associated ROS production and apoptosis reduction in aortic smooth muscle cells

**Figure 5:**
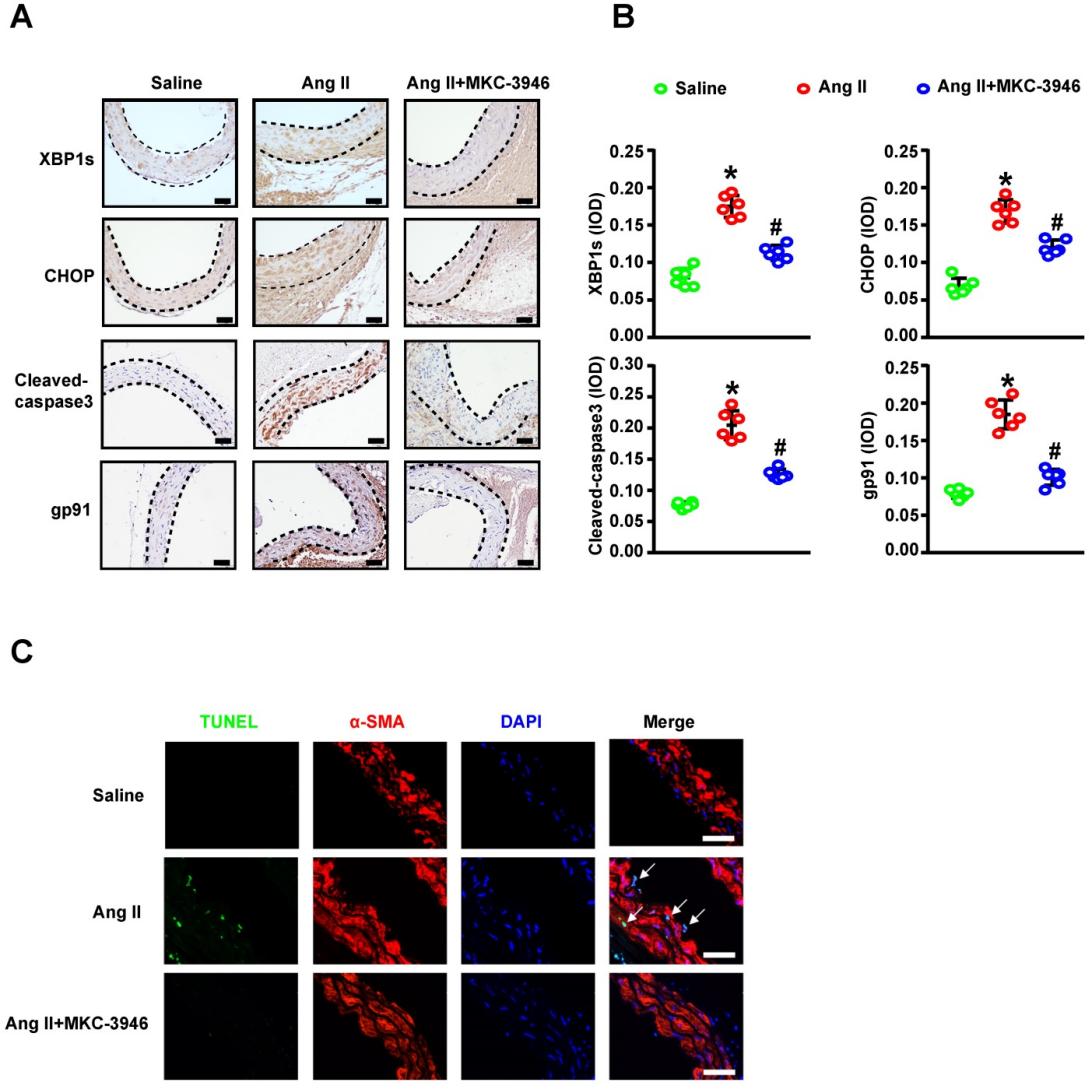
MKC-3946 treatment reduced XBP1s' expression and alleviated apoptosis of aortic smooth muscle cells. **(A)** Representative XBP1s, CHOP, Cleaved-caspase3 and the subunits of NAD(P)H gp91 immunohistochemical staining images of aortae with indicated interventions. **(B)** Quantitative analysis of XBP1s, CHOP, Cleaved-caspase3 and the subunits of NAD(P)H gp91. **(C)** Representative images of aortae showing TUNEL fluorescence in different groups. Green represent TUNEL and blue represent nuclear. Scale bar=50 μm; All data represent the means ± SEM; Scale bar of H&E=500 μm, Scale bar of other images=50 μm; n=6/group; * p < 0.05* vs.* saline group; # *p* < 0.05 *vs.* Ang II group; One-way ANOVA.

Since we have shown that MKC-3946 could reduce the XBP1s expression and subsequent ROS and apoptosis markers triggered by Ang II in cultured VSMCs* in vitro*, we aim to confirm the above findings *in vivo*. Thus, we firstly evaluated the expression levels of XBP1s and CHOP in mouse aortic tissue by Ang II infusion and MKC-3946 treatment. As expected, immunohistochemical staining shows that MKC-3946 treatment markedly reduced the XBP1s expression in the middle layer of the mouse aortae, and CHOP as well. Furthermore, the expression of NAD (P) H subunits gp91 and cleaved-caspase 3 in mouse aortae is higher in Ang II group compared to saline group, while MKC-3946 treatment can significantly reduce their expression (**Figures 5A-B**). Meanwhile, we performed TUNEL staining combined with α-SMA immunofluorescent staining on the aortic sections from different groups of mice and found that Ang II can induce a VSMCs prone apoptosis pattern, while MKC-3946 markedly suppressed it (**Figure 5C**). This means that VSMC apoptosis, which mainly induced by Ang II dependent XBP1s expression, could be blocked by the IRE1α endoribonuclease domain inhibitor MKC-3946* in vivo*.

Taken together, the above results indicate that XBP1s plays a pivotal role in the formation and progression of AD. In detail, Ang II results in ER stress and further induces XBP1s expression. Subsequently, XBP1s translocates into the nucleus, evoking numerous gene transcription, including *CHOP, gp91phox, Cleaved-caspase3, Bax, and Bcl-2*, which play important role in VMSCs apoptosis in the middle layer of the aorta. Pharmacological inhibition of IRE1α endoribonuclease domain by a novel small molecular inhibitor MKC-3946 could reduce the expression of XBP1s and protect against AD in an Ang II induced mouse model by suppressing XBP1s associated ROS production, phenotypic switch and apoptosis in VSMCs, which potentiates its clinical application in the future.

## Discussion

In the current study, we have shown that Ang II elicits overt ER stress in VSMCs, which results in maladaptive of the UPR, this further promotes the tripartite sensors IRE1α, PERK and ATF6 activation. Subsequently, downstream effectors such as XBP1s, ATF4, cleaved ATF6, CHOP, excessive ROS production and phenotypic switch are induced, these together lead to elevated Bax/Bcl-2 ratio, Caspase-3 cleavage and finally VSMC apoptosis. While MKC-3946, a small-molecule IRE1α endoribonuclease domain inhibitor, can ameliorate ER stress and suppress ROS production by inhibiting the XBP1s expression *via* blocking its mRNA splicing *in vitro* and *in vivo*, that results in diminished VSMC apoptosis and retarded AD formation and progression.

Our data indicate that Ang II could be an inducer of ER stress in VSMC, which is consistent with the findings that Ang II treatment increased inflammation and ER stress in adipocytes mainly *via* AT1 receptor 24, 25. Moreover, Chan S *et al.* clearly demonstrated that Ang II could bind to AT1R and induce production of ROS in pancreatic beta cells *via* NADPH oxidase (NOX) and IP3 *via* Gαq/PLCβ. Subsequently, ROS possibly sensitizes the IP3R for subsequent IP3-dependent calcium release from the ER. This causes ER stress and the activation of the UPR, including IRE1α activation and XBP1s expression 25. Furthermore, Ang II has been shown to be a potent stimulator of vascular NAD(P)H oxidase and stimulate ROS production in cultured VSMC 14, excessive ROS triggered ER stress could further promote mitochondrial dysfunction and apoptosis 15. Whether ROS triggers ER stress or ER stress lead to ROS production remains a puzzle 26. Interesting, a dual role of ROS in ER stress signaling has been proposed. In homeostatic state ROS works as signaling intermediates that report ER stress to the UPR, which in turn mitigates ER stress. While under maladaptive state, expression of proteins such as CHOP initiate a secondary rise in ROS, which contributes to unresolved ER stress and cell death 27. Thus, we hypothesize that ER stress and oxidative stress could interact with each other in a context dependent manner.

IRE1α endoribonuclease domain inhibitor MKC-3946 has been investigated in multiple myeloma as well as acute myeloid leukemia. In these tumor cells, IRE1α activation triggered XBP1s expression could drive pro-survival pathways, which results in anti-tumor drug resistance, while MKC-3946 treatment could enhance the tumor cell apoptosis induced by agents like bortezomib, 17-AAG and AS2O3 *in vitro* and* in vivo* 13, 28. These findings reported an opposite effect of MKC-3946 on cell apoptosis. We speculate that in tumor condition, different from normal physiological state, IRE1α activation triggered XBP1s expression is required for the tumor cell growth and survival, but in normal cells, excessive XBP1s production reflect a maladaptive ER stress state, which leads to cell apoptosis. Hence, in our current study MKC-3946 treatment could ameliorate ER stress and suppress ROS in VSMC *in vitro* and *in vivo*.

Although we proposed a vital role of IRE1α-XBP1s signaling pathway in AD formation and progression. We could not exclude other possible related mechanisms. Firstly, hyperactivated IRE1α could increases *TXNIP* mRNA stability *via* miR-17 degradation, elevated TXNIP protein then activates the NLRP3 inflammasome and promote programmed cell death in pancreatic β cell 29. Secondly, IRE1α also has a proapoptotic role, it can bind TRAF2, and activate ASK1 and JNK, thereby leading to caspase-dependent apoptosis 30. Finally, a recent study has shown that under stress condition, downregulation of XBP1u could release its binding partner Forkhead box protein O 4 (FoxO4), which promotes its nuclear translocation. Subsequently, FoxO4 triggered genes expression promoted proinflammatory and proteolytic phenotypic transitioning of VSMC *in vitro*, and resulted in dedifferentiated VSMC and aortic aneurysm formation in a mouse aneurysm model 4. In line with this study, we could also show that Ang II induced excessive XBP1s expression, that could reflect the consumption of XBP1u, while MKC-3946 treatment may increase the XBP1u protein level and protect against VSMC apoptosis *via* the above mentioned XBP1u-FoxO4-myocardin signaling pathway. Further study is required to thoroughly decipher the underlying mechanisms.

When analyzing the human aorta samples, we found that the XBP1s' expression level varies among AD samples. We speculate that there could be several possible reasons as following: 1) Heterogeneity of the patient samples, *e.g.*, genetic background, *etc.*, despite controlled demographic indices; 2) Different disease stage could contribute to these variations; 3) Some comorbidities, life style, environmental factors, *etc.*, could be the potential reasons; 4) Ang II concentration differs among the patients; 5) Sample handling process during operation. However, after careful analysis, we found that the expression of XBP1s is significantly higher in the AD group, which is in agreement with our *in vitro* and *in vivo* results.

In conclusion, the current study revealed the relevant role of IRE1α-XBP1s signaling pathway in AD formation and progression and proved the therapeutic potential of the IRE1α endoribonuclease domain inhibitor MKC-3946 in AD. This study sheds new light on the development of preventive pharmacological treatment for this dreadful disease.

## Figures and Tables

**Table 1:** UPR gene primers for quantitative polymerase chain reaction.

Gene	Forward(5'to3')	Reverse(5'to3')
**m-Srebf2**	GCAGCAACGGGACCATTCT	CCCCATGACTAAGTCCTTCAACT
**m-Rpn1**	ACGAGCCAGCTCTTTCGTTC	GACCGTGAAAAATCTCCCACTT
**m-Cct7**	CCAGTTATCCTGTTGAAAGAGGG	GACCCAGGGTGGTTCTTACA
**m-Htra2**	TAGGACCCCGGATCTCTGG	GACCCCAACCCCACAACAG
**m-Creb3**	AAGGCTCCGCTGGACTTAGA	TGTGGAAGGGAGTAGTTGTGA
**m-Bax**	TGAAGACAGGGGCCTTTTTG	AATTCGCCGGAGACACTCG
**m-Nucb1**	GACACGGGCCTGTACTACC	CCATCTGTCTCTAGCACGTTGA
**m-Ganab**	TGGCCGTGATGACAACAGTG	GCGCGAGACTAACTTTATCCGA
**m-Canx**	ATGGAAGGGAAGTGGTTACTGT	GCTTTGTAGGTGACCTTTGGAG
**m-Erp44**	TGCGGTCTTCCTGTCTTTAGC	AACGACACCAGTCAGCATAAAA
**m-Edem1**	AGTCAAATGTGGATATGCTACGC	ACAGATATGATATGGCCCTCAGT
**m-Vcp**	CGACCCAATCGGTTAATTGTTGA	AGCTTCCCGTCTTTTCTTTCC
**m-Dnajb9**	CTCCACAGTCAGTTTTCGTCTT	GGCCTTTTTGATTTGTCGCTC
**m-Dnajb3**	CGAGAGGCGGTTCAAGCAG	CCCCCGAAGAAGTCGAAGG
**m-Herpud1**	GCAGTTGGAGTGTGAGTCG	TCTGTGGATTCAGCACCCTTT
**m-Hspa2**	GCGTGGGGGTATTCCAACAT	TGAGACGCTCGGTGTCAGT
**m-Rnf5**	CAAGAATGCCCGGTGTGTAAA	GGGTGGAGTTTTCAATCTGGGA
**m-Sec62**	AAGGCTGTAGCCAAATATCTTCG	CCACAGACTCCCTGGTTGTAAAT
**m-Sec63**	TGACAGCGGGAACACCTTC	GGCTGGGGTTTTAATAACCGTA
**m-Ufd1I**	ACCGCTTCTCCACGCAGTAC	AGTTGATCGAGGGCTGAGGG
**m-GAPDH**	TGGATTTGGACGCATTGGTC	TTTGCACTGGTACGTGTTGAT

## Abbreviations

AD: aortic dissection
EVAR: endovascular aortic repair
VSMC: vascular smooth muscle cell
ER stress: Endoplasmic reticulum stress
UPR: unfolded protein response
PERK: protein kinase R-like ER kinase
ATF6: activating transcription factor 6
IRE1α: inositol-requiring enzyme 1α
CHOP: C/EBP-homologous protein
XBP1s: spliced X-box Binding Protein 1
XBP1u: unspliced X-box Binding Protein 1
ROS: reactive oxygen species
Ang II: Angiotensin II
BAPN: β-aminopropionitrile

## References

[1] Shan XS, Dai HR, Zhao D, Yang BW, Feng XM, Liu H (2021). Dexmedetomidine reduces acute kidney injury after endovascular aortic repair of Stanford type B aortic dissection: A randomized, double-blind, placebo-controlled pilot study. J Clin Anesth.

[2] Levy D, Goyal A, Grigorova Y, Farci F, Le JK (2021). Aortic Dissection. StatPearls. Treasure Island (FL).

[3] Wu D, Shen YH, Russell L, Coselli JS, LeMaire SA (2013). Molecular mechanisms of thoracic aortic dissection. J Surg Res.

[4] Zhao G, Fu Y, Cai Z, Yu F, Gong Z, Dai R (2017). Unspliced XBP1 Confers VSMC Homeostasis and Prevents Aortic Aneurysm Formation via FoxO4 Interaction. Circ Res.

[5] Jia LX, Zhang WM, Zhang HJ, Li TT, Wang YL, Qin YW (2015). Mechanical stretch-induced endoplasmic reticulum stress, apoptosis and inflammation contribute to thoracic aortic aneurysm and dissection. J Pathol.

[6] Navas-Madronal M, Rodriguez C, Kassan M, Fite J, Escudero JR, Canes L (2019). Enhanced endoplasmic reticulum and mitochondrial stress in abdominal aortic aneurysm. Clin Sci (Lond).

[7] Yoshida H, Matsui T, Yamamoto A, Okada T, Mori K (2001). XBP1 mRNA is induced by ATF6 and spliced by IRE1 in response to ER stress to produce a highly active transcription factor. Cell.

[8] Lee AH, Iwakoshi NN, Glimcher LH (2003). XBP-1 regulates a subset of endoplasmic reticulum resident chaperone genes in the unfolded protein response. Mol Cell Biol.

[9] Zeng L, Xiao Q, Chen M, Margariti A, Martin D, Ivetic A (2013). Vascular endothelial cell growth-activated XBP1 splicing in endothelial cells is crucial for angiogenesis. Circulation.

[10] Zeng L, Zampetaki A, Margariti A, Pepe AE, Alam S, Martin D (2009). Sustained activation of XBP1 splicing leads to endothelial apoptosis and atherosclerosis development in response to disturbed flow. Proc Natl Acad Sci U S A.

[11] Margariti A, Li H, Chen T, Martin D, Vizcay-Barrena G, Alam S (2013). XBP1 mRNA splicing triggers an autophagic response in endothelial cells through BECLIN-1 transcriptional activation. J Biol Chem.

[12] Liu W, Zhang W, Wang T, Wu J, Zhong X, Gao K (2019). Obstructive sleep apnea syndrome promotes the progression of aortic dissection via a ROS- HIF-1alpha-MMPs associated pathway. Int J Biol Sci.

[13] Mimura N, Fulciniti M, Gorgun G, Tai YT, Cirstea D, Santo L (2012). Blockade of XBP1 splicing by inhibition of IRE1alpha is a promising therapeutic option in multiple myeloma. Blood.

[14] Montezano AC, Nguyen Dinh Cat A, Rios FJ, Touyz RM (2014). Angiotensin II and vascular injury. Curr Hypertens Rep.

[15] Yang Y, Wang G, Wu W, Yao S, Han X, He D (2018). Camalexin Induces Apoptosis via the ROS-ER Stress-Mitochondrial Apoptosis Pathway in AML Cells. Oxid Med Cell Longev.

[16] Clement M, Chappell J, Raffort J, Lareyre F, Vandestienne M, Taylor AL (2019). Vascular Smooth Muscle Cell Plasticity and Autophagy in Dissecting Aortic Aneurysms. Arterioscler Thromb Vasc Biol.

[17] Yang K, Ren J, Li X, Wang Z, Xue L, Cui S (2020). Prevention of aortic dissection and aneurysm via an ALDH2-mediated switch in vascular smooth muscle cell phenotype. Eur Heart J.

[18] Wang W, Liu Q, Wang Y, Piao H, Zhu Z, Li D (2021). LINC01278 Sponges miR-500b-5p to Regulate the Expression of ACTG2 to Control Phenotypic Switching in Human Vascular Smooth Muscle Cells During Aortic Dissection. J Am Heart Assoc.

[19] Frismantiene A, Philippova M, Erne P, Resink TJ (2018). Smooth muscle cell-driven vascular diseases and molecular mechanisms of VSMC plasticity. Cell Signal.

[20] Lai CH, Chang CW, Lee FT, Kuo CH, Hsu JH, Liu CP (2020). Targeting vascular smooth muscle cell dysfunction with xanthine derivative KMUP-3 inhibits abdominal aortic aneurysm in mice. Atherosclerosis.

[21] Peng H, Zhang K, Liu Z, Xu Q, You B, Li C (2018). VPO1 Modulates Vascular Smooth Muscle Cell Phenotypic Switch by Activating Extracellular Signal-regulated Kinase 1/2 (ERK 1/2) in Abdominal Aortic Aneurysms. J Am Heart Assoc.

[22] Badran A, Nasser SA, Mesmar J, El-Yazbi AF, Bitto A, Fardoun MM (2020). Reactive Oxygen Species: Modulators of Phenotypic Switch of Vascular Smooth Muscle Cells. Int J Mol Sci.

[23] Li B, Wang Z, Hu Z, Zhang M, Ren Z, Zhou Z (2017). P38 MAPK Signaling Pathway Mediates Angiotensin II-Induced miR143/145 Gene Cluster Downregulation during Aortic Dissection Formation. Ann Vasc Surg.

[24] Menikdiwela KR, Ramalingam L, Allen L, Scoggin S, Kalupahana NS, Moustaid-Moussa N (2019). Angiotensin II Increases Endoplasmic Reticulum Stress in Adipose Tissue and Adipocytes. Sci Rep.

[25] Chan SMH, Lau YS, Miller AA, Ku JM, Potocnik S, Ye JM (2017). Angiotensin II Causes beta-Cell Dysfunction Through an ER Stress-Induced Proinflammatory Response. Endocrinology.

[26] Malhotra JD, Kaufman RJ (2007). Endoplasmic reticulum stress and oxidative stress: a vicious cycle or a double-edged sword?. Antioxid Redox Signal.

[27] Ochoa CD, Wu RF, Terada LS (2018). ROS signaling and ER stress in cardiovascular disease. Mol Aspects Med.

[28] Sun H, Lin DC, Guo X, Kharabi Masouleh B, Gery S, Cao Q (2016). Inhibition of IRE1alpha-driven pro-survival pathways is a promising therapeutic application in acute myeloid leukemia. Oncotarget.

[29] Lerner AG, Upton JP, Praveen PV, Ghosh R, Nakagawa Y, Igbaria A (2012). IRE1alpha induces thioredoxin-interacting protein to activate the NLRP3 inflammasome and promote programmed cell death under irremediable ER stress. Cell Metab.

[30] Urano F, Wang X, Bertolotti A, Zhang Y, Chung P, Harding HP (2000). Coupling of stress in the ER to activation of JNK protein kinases by transmembrane protein kinase IRE1. Science.

